# Design considerations in a clinical trial of a cognitive behavioural intervention for the management of low back pain in primary care: Back Skills Training Trial

**DOI:** 10.1186/1471-2474-8-14

**Published:** 2007-02-22

**Authors:** Sarah E Lamb, Ranjit Lall, Zara Hansen, Emma J Withers, Frances E Griffiths, Ala Szczepura, Julie Barlow, Martin R Underwood, the Back Skills Training Trial (BeST) Team

**Affiliations:** 1 Warwick Clinical Trials Unit, Health Sciences Institute, Warwick Medical School, University of Warwick, Coventry CV4 7AL, UK; 2 Kadoorie Critical Care Research Centre, University of Oxford, Oxford OX3 9DU, UK; 3 Primary Health Care Studies, Health Sciences Institute, Warwick Medical School, University of Warwick, Coventry CV4 7AL, UK; 4 Clinical Sciences Institute, Warwick Medical School, University of Warwick, Coventry CV4 7AL, UK; 5 Interdisciplinary Research Centre in Health, Faculty of Health & Life Sciences, Coventry University CV1 5FB, Coventry, UK; 6 Centre for Health Sciences, Queen Mary University of London E1 4NS, UK

## Abstract

**Background:**

Low back pain (LBP) is a major public health problem. Risk factors for the development and persistence of LBP include physical and psychological factors. However, most research activity has focused on physical solutions including manipulation, exercise training and activity promotion.

**Methods/Design:**

This randomised controlled trial will establish the clinical and cost-effectiveness of a group programme, based on cognitive behavioural principles, for the management of sub-acute and chronic LBP in primary care. Our primary outcomes are disease specific measures of pain and function. Secondary outcomes include back beliefs, generic health related quality of life and resource use. All outcomes are measured over 12 months. Participants randomised to the intervention arm are invited to attend up to six weekly sessions each of 90 minutes; each group has 6–8 participants. A parallel qualitative study will aid the evaluation of the intervention.

**Discussion:**

In this paper we describe the rationale and design of a randomised evaluation of a group based cognitive behavioural intervention for low back pain.

## Background

Low back pain (LBP) is a major public health problem [1,2]. In the UK, the annual period prevalence of LBP is approximately 37% [3,4]. A study conducted in the UK found that 75% of people with LBP who consulted their general practitioner (GP) still had symptoms one year later; 30% had developed persistent disabling LBP [5,6]. The direct health care costs associated with LBP in 1998 were £1628 million; the majority of this was spent on physiotherapy and general practice [1].

Since the early 1990s there has been a change in the emphasis of LBP treatment with strong discouragement of bed rest and encouragement of physical activity becoming an orthodox approach. This active management strategy forms the core of all international guidelines for the management of acute (< 3 months) LBP [7-12]. Less attention has been paid to the management of chronic low back pain. The active management approach is sometimes supplemented with patient education materials. In the UK, 'The Back Book' is advocated [13,14]. Evidence to support other physical treatments is weak [15]. A recent UK study found no difference between six sessions of physical therapy and a single session of active management supplemented by 'The Back Book' [16] There are no recommendations relating to the use of psychologically based treatments [17].

A number of studies indicate that cognitive behavioural approaches (CBA) may be beneficial in the management of sub-acute and chronic LBP [17-22]. However, none of the trials to date has been of sufficient size or duration to determine long-term clinical and there has been little attention paid to the cost effectiveness of CBA.

Applications of CBA for LBP have varied in content and method of delivery [23]. In the UK, the first applications of CBA were in-patient pain management programmes for very chronic low back pain. CBA appears moderately effective in this context [18], but the effect on people presenting in general practice, often with less severe symptoms, is unknown. Although not formally reported, pain management programmes are expensive because of the intensity of intervention and high staff costs. Less expensive CBA, such as short group programmes led by a nurse or therapist, would appear highly applicable to general practice, but have not been widely implemented or studied in such settings. Potential advantages include; preventing chronic disability, increasing physical and psychosocial functioning in patients with disability due to low back pain, and decreasing inappropriate health care utilisation. In other chronic conditions the social interactions and comparisons that occur in a group-based intervention have been identified as potentially important mediators of the therapeutic effect [24]. The Back Skills Training Trial (BeST) has been designed to capture and evaluate these effects using a combination of qualitative and quantitative methodologies.

There is a need to establish if group based CBA is an effective approach in the management of sub-acute and chronic LBP, and if baseline characteristics are important predictors of treatment response. We will determine the effectiveness of adding a group based, professionally led CBA for LBP to active management in general practice on

• LBP related pain and disability

• time lost from occupational activity

• fear avoidance beliefs

• the use of further medical, rehabilitation, surgical or alternative treatments for LBP

• generic health related quality of life

• health service costs.

The target population are individuals with low back pain of at least moderate troublesomeness and of at least six weeks duration. The effects will be monitored over a 12-month period. Evaluation will include an appropriate method of cost appraisal which will consider both the health and societal perspective.

## Methods/Design

### Setting

Around 98% of the UK population is registered with a general practitioner. We are recruiting participants from 97 general practices, with a total list size of 700,000 patients in seven localities (Primary Care Trusts) across England; South Warwickshire, North Warwickshire, Coventry, Solihull, North Norfolk, Southern Norfolk, Norwich, Broadland, Langbaugh, Heart of Birmingham, and South Birmingham. The population in these localities is broadly representative of the population of England. Participants from practices in each locality are able to attend the same treatment centre ensuring sufficient numbers to sustain the group sessions without making participant travel burdensome.

### Ethical approval

West Midlands Multi-Centred Research Ethics Committee, Birmingham UK (MRC/03/7/04) provided the ethical review and approval.

### Study Population

Potential participants who have consulted their general practice with back pain in the last six months are identified by searching the practice computerised medical record or are identified by practice clinical staff when they attend the practice. They are sent an invitation letter and eligibility questionnaire by post. Those people who indicate a willingness to participate, and fulfil the first stage eligibility criteria are invited to an initial interview with a research nurse at which there are further eligibility checks, and potential participants are provided with a detailed explanation of the study purpose and procedures. At a second appointment, at least one week later, informed consent is obtained, baseline data are collected, the participant is randomised and the 'active management approach' to managing back pain is reinforced. This includes providing all participants with a copy of 'The Back Book'.

### Eligibility criteria

Inclusion criteria are LBP of at least moderate troublesomeness and of at least six weeks duration; age greater than 18 years; willing and able to give informed consent and to understand/speak English. People are ineligible if they have been managed previously in a cognitive behavioural programme; have "Red Flags" i.e. factors associated with serious LBP pathology (including cauda equina symptoms, systemic illness (including cancer, HIV, fever); widespread neurological problems, severe unremitting night-time pain, violent trauma (fall from height, RTA), unexplained weight loss); or have severe psychiatric or personality disorders. There is consensus that CBA and related interventions are unnecessarily intensive for people who suffer an isolated acute or minor episode of LBP, in whom symptoms resolve quickly and pose no on-going problem [25]. We are seeking to recruit participants with at least moderately troublesome sub-acute or chronic low back pain [26]. Measuring 'troublesomeness'/'bothersomeness' is a simple criterion for determining overall symptom burden [27,28].

### Baseline Assessment

Data are collected using a standard pro forma administered by a specially trained research nurse. These include age, gender, ethnicity, educational attainment, general health status, duration of LBP, symptoms, recent treatment history, anxiety and depression (using the Hospital Anxiety and Depression Scale [29]) and employment status. In addition participants complete the baseline (pre-intervention) versions of the outcome measure package (see Table 1).

**Table 1:** Outcomes measures

	Domain	Measures	Time points (months)
Primary	Pain & Disability	Roland & Morris Questionnaire (Roland 1983)	0, 3, 6, 12
	Pain	Von Korff Scale (Von Korff 1992)	0, 3, 6, 12
Secondary	Occupational and other limitations	Numbers of days off work, reduced activity and bed rest	0, 3, 6, 12
	Health related quality of life inc physical & mental health	Short Form 12 version 2 (Ware 1996)	0, 3, 6, 12
	Back Pain Beliefs	Fear avoidance scale (1^st ^five items only)* (Waddell 1993)	0, 3, 6, 12
	Self-efficacy	Pain self-efficacy questionnaire (Nicholas 2006)	0, 3, 6, 12
	Satisfaction with treatment	Single item rating of satisfaction with treatment (Deyo et al 1998)	12
Economic analysis	Resource Use	Resource use questionnaire	6, 12
	Health related quality of life; time trade off score	EQ-5D (health utility) (EuroQol Group 1990)	0, 6, 12

### Treatment allocation

**Figure 1:**
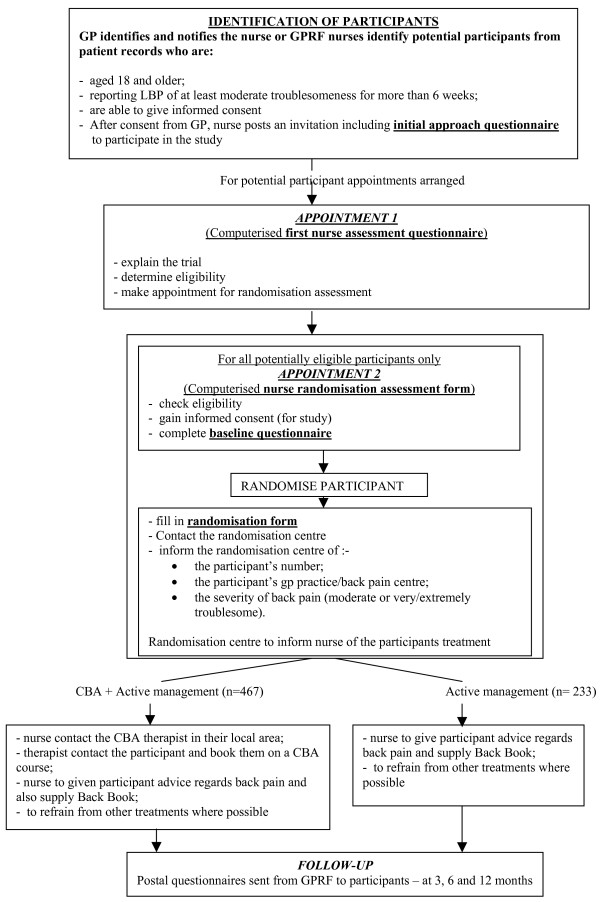
Flow diagram of the study

Following completion of the baseline assessment, the research nurse completes a randomisation form and telephones a central randomisation office to obtain the treatment allocation. A minimisation algorithm is used to ensure that, within each treatment arm, near equal numbers of participants are entered from each centre within the differing levels of troublesome back pain (moderately or very/extremely troublesome). The nurse provides the advice component of the intervention to all study participants, and for participants randomised to CBA, sends a notification letter to the local service provider. The service provider then contacts the participant to arrange a day/time to start the CBA treatment. Fig 1

### Experimental intervention

Back Skills Training is a 'complex' intervention, comprising a number of components that may act both independently and inter-dependently [30]. The components are;

(i) education to counter unhelpful beliefs about LBP and to highlight the importance of appropriate levels of activity;

(ii) use of cognitive re-structuring techniques to counter unhelpful beliefs;

(iii) training on goal setting, baseline setting, and pacing for incrementally increasing activities;

(iv) specific focus on fear avoidance and attentional effects on pain;

(v) techniques for self-management of pain especially in flare-ups.

These components are delivered using a cognitive behavioural model and focus on group problem solving to increase self-efficacy. The treatment sessions are delivered to groups of approximately 6–8 patients, using six weekly sessions of 90 minutes length each. The rationale and the components will be described in detail elsewhere. A participant is deemed to have received the intervention if they attend the baseline assessment, and three of the six follow-up sessions. Therapists and nurses receive a one and half day training programme including basic principles of the cognitive behavioural approach, questioning, facilitation of groups, pacing, coping, relaxation, exercise, and pain management. Training and support are provided by a qualified cognitive behavioural therapist and physiotherapist. The intervention is structured around standardised sessions and supplemented by a participant workbook.

### Other treatments

All participants receive a simple active management intervention including a copy of 'The Back Book' [13]. The important components of the active management strategy are emphasised by the research nurse; namely,

• Encouragement to remain active and resist bed rest.

• Advice on appropriate methods of pain control.

Before starting the study the research nurses are trained in delivery of the active management strategy supplemented by 'The Back Book' [13]. Participants are asked to act on the guidance. Participants can if they wish seek further assistance from their GP or chosen back care provider. GPs are able to refer patients to any other services that they consider appropriate.

### Outcome measures

The hypothesised benefits of a CBA are;

a) improvements in pain and LBP disability,

b) improved tolerance to pain, increased self-efficacy and decreased depression,

c) improvements in overall quality of life

d) an increase in activity, particularly in those activities that are avoided due to fear of pain or symptoms,

e) a shift toward self-management of LBP symptoms and disability,

f) a reduction in use of self-paid and/or NHS treatments.

The outcome measures (shown in Table 1) have been selected to cover these domains, and to maximise generalisability, and have utilised the outcome data set recommended by the International Low Back Pain Forum where appropriate [2]. The primary outcome is the Roland and Morris Questionnaire which (RMDQ) [31] is one of the most widely used measures for LBP in primary care. It has acceptable reliability [32,2,33] but concerns are emerging about its scalability and sensitivity to clinically important change [34]. Therefore, have a second primary outcome with the modified Von Korff Scale [35] and we will undertake additional analyses to compare the psychometric properties of the two measures. The secondary outcome measures are:

(i) the number of days (defined as greater than 1/2 a day) participants have had to cut down on normal activity in the preceding four weeks;

(ii) number of days participants had time off work because of low back pain or leg pain (sciatica);

(iii) psychological measures as captured on the fear avoidance beliefs questionnaire, pain self-efficacy

(iv) health-related quality of life as assessed using the SF-12 Version 2;

(v) satisfaction with treatment measured using a standardised and recommended single-item question [2] – 'How satisfied are you with the treatment you received?'. The response is measured on a five point Likert scale ranging from very dissatisfied to very satisfied.

If the intervention is successful participants should be able to utilise cognitive skills to manage symptoms over a prolonged period; so we will measure the potential benefits over a 12-month follow up period. The timing of various assessments is shown in Table 1.

### Sample size

The sample size has been estimated using well-documented methods [36], with careful consideration of the practicalities to ensure that sufficient numbers of participants are randomised within an acceptable time frame. We originally planned a 1:1 randomisation ratio between CBA and usual care. Early in the trial we recognised that a 2:1 randomisation (in favour of CBA) had distinct advantages in ensuring that sufficient numbers of patients are randomised to sustain the group sessions. A randomisation balance of 2:1 can be adopted with inconsequential loss of power, but further imbalances necessitate an increase in study size. From a list size of 100,000 (approximating to one location), with twice as many participants being randomised to the intervention group, we estimated the yield of participants randomised to the group sessions was 1.4 per week, allowing us to recruit eight participants to a new group starting every six weeks. This estimate was based on data from the UK BEAM trial [37].

The potential impact of the group effect was also considered in the sample size estimation. In this trial, other effects include the individual practitioner treating several groups, regions and organisations. We perceive that the most potent of the cluster effects is the group effect, and is the only effect to have been considered formally in the sample size estimation (using methods described by [38]).

The sample size target for the trial is 700 to detect a moderate effect size, and a difference between the groups of approximately 1.8 RDQ points, assuming a standard deviation of 4.27 points (giving an effect size of 0.42). Approximately 233 participants will be randomised to active management alone and 467 participants on the active management + CBA arm of the trial. The sample size has been inflated to account for an inter-cluster correlation co-efficient of 0.01 (based on UK – BEAM [37] and incorporates a loss to follow up of 25%. We have used a power of 90% and p < 0.01 [39]. Effect sizes of approximately 0.4 are considered to be clinically worthwhile for back pain interventions [23]. The sample size requirement will be reviewed by an independent data monitoring committee at approximately the mid point of data accumulation.

### Data analysis

Primary analyses will be by intention-to-treat, i.e. patients will be analysed in the groups to which they were randomised, regardless of the treatment that they may have received. The main study outcomes will be summarised as the Area under the Curve (AUC) over the 12 month follow up period. The AUC will be calculated by summing areas under the graph between each pair of consecutive observations for an individual. Thus the AUC is a weighted average of the outcome scores at each individual time point weighted by the time between the observations. Mean/median (depending on the distribution of the data) AUC between the two treatment groups will then be compared by a two independent sample t-test/Wilcoxon rank sum test and include reporting of the 95% confidence intervals for the mean/median difference in the AUCs. One of the advantages of the AUC method is that a sensitivity analysis may be easily performed to investigate the effect of missing data. In the case of large missing data, imputation techniques will be considered. In addition outcomes will be reported separately for the 3 month time point to characterise the early response to treatment. Multi-level modelling will be used to estimate group, therapist and other effects as appropriate.

### Sub-group analyses

The potential biases inherent in undertaking multiple sub-group analyses are well recognised [40]. However, the BeST trial offers a unique opportunity to generate hypotheses about the profile of patients most likely to benefit from group based CBA. The most scientifically robust method of sub-group analysis is a test of interaction between treatment and outcome that has been appropriately powered. It is widely recognised that powering a sub group analysis can dramatically increase sample size requirement. A rough rule is that detection of interactions approximately twice the size of the main effect requires no increase in the sample size, provided that the sub-groups are of equal size, the sub-group comparisons are limited and pre-specified, and the results are considered hypotheses generating as opposed to confirmatory [40]. We will report two pre-specified analyses alongside the main trial results, namely a comparison of treatment effect in those groups

i) with sub-acute versus chronic low back pain at study entry.

ii) with moderately versus very/extremely troublesome back pain at study entry.

iii) With high versus low fear avoidance at study entry.

These comparisons assume that detection of large effects, and that sub-groups are of roughly equal size.

## Discussion

### Estimates of cost consequences

LBP has a range of costs and consequences across healthcare and patients. Once data collection is complete, the costs and consequences of each treatment arm will be compared from a societal as well as from a health care perspective. The cost of each treatment strategy is being determined prospectively and includes staff time, overheads, equipment and transport. We are administering a closed structure questionnaire to participants during the follow up period (see Table 1), to ascertain whether participants have had additional NHS or private treatment for their LBP and whether this was paid for by the individual or insurance provider. Participants are asked about medication over the preceding 4 weeks, and to distinguish between prescription and over-the-counter expenses. Patient self-reported information on service use has been shown to be accurate in terms of intensity of use of different services [41].

NHS service use associated with each treatment arm is collected across study sites. The resource use estimates will be complemented by other national sources. In order to value the cost of these services, it will be assumed that average costs reasonably reflect the long run marginal costs of provision of services. The use of primary care and hospital services will be costed from a variety of sources, including the finance departments of the hospitals, PCTs concerned and national sources [42]. There will be uncertainties in many of the statistical estimates (e.g. mean number of GP visits) and certain assumptions (e.g. the average cost per visit). A careful analysis of the sensitivity of any observed cost differences between areas will be undertaken, based on the confidence intervals around the statistical estimates and alternative assumptions. Multi-way analysis will be undertaken along with an estimate of critical values of key variables that can reverse the result. Full economic evaluation will be performed based on a comparative assessment of the marginal costs and outcomes of the two treatment regimes used. A cost-utility analysis will present the incremental cost of the extra benefit gained; costs for any improvement of the health status index (EQ-5D) will be calculated over time. This will be done both in summary form in terms of incremental cost per QALY, and also using a 'disaggregated' approach where the extra costs are presented alongside the outcome gains in terms of improvements in pain, physical activity, mental well-being etc. In all these analyses, the uncertainties in the cost and outcomes data will be incorporated into a sensitivity analysis. Resource implications will be combined with estimates of effectiveness derived for the two components of the trial [43].

### Qualitative study

A parallel qualitative study is designed to increase our understanding of the participant's experience of back pain and treatment and to provide detailed descriptive data to inform transferability of the trial outcomes to other individuals, context and similar interventions. A researcher experienced in social science methodology is conducting in-depth interviews with approximately 30 individuals (15 from each treatment arm). Sampling is for diversity of age, severity of disability at entry into the trial and fear avoidance. The interviews are following a semi-structured approach, and are audio-taped and transcribed. Data analysis will use the framework approach [44]). Qualitative data will be collected on two occasions: after randomisation and 12 months after treatment.

We have presented the rationale and design of a trial to evaluate a complex intervention to improve low back pain. Trial recruitment has commenced and is due to close in March 2007. Follow up will continue until June 2008, and results will be finalised for publication by January 2009.

## Competing interests

The author(s) declare that they have no competing interests.

## Pre-publication history

The pre-publication history for this paper can be accessed here:


